# The significance of compliance and persistence in the treatment of diabetes, hypertension and dyslipidaemia: a review

**DOI:** 10.1111/j.1742-1241.2007.01630.x

**Published:** 2008-01

**Authors:** J A Cramer, Á Benedict, N Muszbek, A Keskinaslan, Z M Khan

**Affiliations:** 1 Yale University School of Medicine, West Haven CT, USA; 2 United BioSource Corporation Budapest, Hungary and London, UK; 3 Novartis Pharma AG Basel, Switzerland; 4 Novartis Pharmaceuticals Corporation East Hanover, NJ, USA

## Abstract

**Objectives:**

To review studies of patient compliance/persistence with cardiovascular or antidiabetic medication published since the year 2000; to compare the methods used to measure compliance/persistence across studies; to compare reported compliance/persistence rates across therapeutic classes and to assess whether compliance/persistence correlates with clinical outcomes.

**Methods:**

English language papers published between January 2000 and November 2005 investigating patient compliance/persistence with cardiovascular or antidiabetic medication were identified through searches of the MEDLINE and EMBASE databases. Definitions and measurements of compliance/persistence were compared across therapeutic areas using contingency tables.

**Results:**

Of the 139 studies analysed, 32% focused on hypertension, 27% on diabetes and 13% on dyslipidaemia. The remainder covered coronary heart disease and cardiovascular disease (CVD) in general. The most frequently reported measure of compliance was the 12-month medication possession ratio (MPR). The overall mean MPR was 72%, and the MPR did not differ significantly between treatment classes (range: 67–76%). The average proportion of patients with an MPR of > 80% was 59% overall, 64% for antihypertensives, 58% for oral antidiabetics, 51% for lipid-lowering agents and 69% in studies of multiple treatments, again with no significant difference between treatment classes. The average 12-month persistence rate was 63% and was similar across therapeutic classes. Good compliance had a positive effect on outcome in 73% of the studies examining clinical outcomes.

**Conclusions:**

Non-compliance with cardiovascular and antidiabetic medication is a significant problem, with around 30% of days ‘on therapy’ not covered by medication and only 59% of patients taking medication for more than 80% of their days ‘on therapy’ in a year. Good compliance has a positive effect on clinical outcome, suggesting that the management of CVD may be improved by improving patient compliance.

**Review Criteria**

Studies evaluating adherence, persistence and/or compliance with cardiovascular or antidiabetic medication were identified through searches of the MEDLINE and EMBASE databases. A manual search of reference lists from retrieved papers was also performed. Prespecified parameters from relevant papers were recorded and analysed numerically.

**Message for the Clinic**

A literature review of 139 studies reporting compliance data showed that non-compliance with cardiovascular and antidiabetic medication is a significant problem. Only 63% of patients continue with their medication for a year and patients only take their medication for 72% of the time, yet in 73% of studies good compliance had a positive effect on clinical outcomes. Encouraging patients to comply with their treatment regimens could do much to improve the clinical management of cardiovascular disease.

## Introduction

Hypertension, dyslipidaemia and diabetes are well-known risk factors for cardiovascular disease (CVD), which is a leading cause of death and disability worldwide (1–5). Large-scale clinical trials have shown that pharmacological treatment can reduce the morbidity and mortality associated with CVD and that long-term or lifelong treatment is often indicated (6–9).

According to the World Health Organization, non-compliance with long-term medication for conditions such as hypertension, dyslipidaemia and diabetes is a common problem that leads to compromised health benefits and serious economic consequences in terms of wasted time, money and uncured disease (10). In addition, a recent editorial referred to the overwhelming evidence for a decrease in morbidity and mortality with the use of antihypertensive therapy, and concluded that the greatest potential for improving control of hypertension lies in improving patient compliance (11).

Compliance with medication has become a topic of much research, and various interventions have been proposed to improve patient compliance. However, it has proved difficult to compare studies of compliance because of a lack of standard terminology and methodology. Two recent Cochrane reviews of interventions aimed at improving compliance with lipid-lowering and antihypertensive treatments found ‘substantial heterogeneity’ in the measures of compliance used and therefore did not attempt to combine specific studies (12, 13).

A literature review of research into patient compliance with antihypertensive, lipid-lowering or oral antidiabetic medications was therefore performed to aid current understanding of the medical significance of patient compliance in the treatment of CVD. The aims of the study were to review original research papers measuring compliance and/or persistence with antihypertensives (AHTs), lipid-lowering therapies (LLTs) and oral antidiabetics (OADs) published between 2000 and 2005; to compare the methodology used within the studies to measure compliance/persistence; to compare the reported compliance/persistence rates at the study level across the three treatment areas and to assess whether compliance/persistence correlates with clinical outcomes.

## Methods

### Searches

Searches for relevant research reports were conducted using the MEDLINE and EMBASE databases. The search terms used were: cardiovascular, hypertens*, hyperlipid*, dyslipid*, blood pressure, diabet*, adherence, persistence and compliance. A manual search of the reference lists from retrieved papers was also performed to identify further relevant studies.

### Selection criteria

Studies were deemed relevant if they were English language, human, original research studies published between January 2000 and November 2005; if they involved patients with CVD or diabetes; if they examined compliance and/or persistence with pharmaceutical interventions (even if the primary objective was not to measure compliance); and if they provided a numeric measure of compliance or persistence with an adequate description of the methodology used. Posters were included only if available. Trials of clinical efficacy were not included unless they specifically investigated compliance. As the objective of any clinical trial is to maintain compliance at the highest possible level and as it is necessary to adhere to a protocol, such studies were not considered to be relevant to this assessment of compliance because they would be biased and skewed towards high compliance. The search was restricted to the 5-year period 2000–2005 to capture recent studies.

Studies were excluded from analysis if the study design and methods for calculating compliance/persistence were not appropriately described; if no numeric value for compliance/persistence was reported; if they examined non-compliance with antiplatelets, aspirin, digoxin, insulin, non-pharmaceutical therapies or treatment guidelines; and if they were reviews of earlier research papers, letters to the editor, commentaries or conference abstracts.

### Data extraction

Parameters extracted from the studies included study design, country of study, number of patients, mean age of patients (weighted averages for studies with multiple treatment arms), mean study length (median if mean not available or time-frame of data collection if mean and median not available), definition of compliance or persistence, unit of measure of compliance or persistence and type of funding. No limits were set on the number of patients or the study length. Studies supported by industry (such as pharmaceutical companies, managed care organisations and consultancies) were identified, but unrestricted grants from pharmaceutical companies were considered to be non-industry funded.

Patients were classified as having hypertension, diabetes, dyslipidaemia, CVD or coronary heart disease (CHD). Myocardial infarction (MI) and heart failure were included under CHD, while stroke/transient ischaemic attack and other unspecified cardiovascular conditions were classified under CVD. Treatments were divided into AHTs, OADs and LLTs. Studies examining two or three therapeutic classes were categorised as ‘multiple treatment’.

### Compliance definitions, measurements and data sources

**Figure 1:**
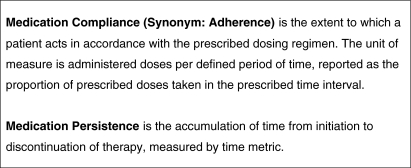
Definitions of medication compliance and persistence proposed by the Issues and Methods Definitions Working Group of the Medication Compliance and Persistence Special Interest Group (15)

Two common measures of compliance are adherence (sometimes used as a synonym for compliance) and persistence. Adherence refers to the proportion of pills taken within a specific time interval and persistence refers to the continuing use (in time) of the prescribed therapy (14). To aid future research into compliance, the Medication Compliance and Persistence Special Interest Group (MCP SIG) of the International Society for Pharmacoeconomics and Outcomes Research has proposed standard international definitions of medication compliance (adherence) and persistence (Figure 1) (15).

A commonly used measure of compliance is the mean medication possession ratio (MPR). This is usually defined as the number of days of treatment dispensed divided by the number of days between prescription refills (excluding the last prescription) (16). For example, a patient who receives daily treatment and is prescribed 90 days of medication but does not refill the prescription for a further 10 days has an MPR of 90/100 or 90%. Another commonly used measure of compliance is the percentage of patients with an MPR of more than 80% over a certain period of time, although the rationale for this cut-off point is often not justified and it is not necessary to use categorical rather than continuous data. A commonly used measure of persistence is the percentage of patients who are persistent with treatment at 1 year.

For this review, definitions of compliance and persistence, and their method of measurement, were recorded. Data on the MPR or the percentage of patients with an MPR of more than 80% at 1 year were extracted and analysed numerically. The percentage of patients persisting with treatment after 1 year was also analysed. All measures were classified as either continuous (able to take on any value, such as MPR) or discrete (limited to specific values). One compliance rate was calculated for each study. In studies with multiple treatment arms, population-weighted averages were used.

The sources of prospectively collected compliance data in the studies were also identified and classified as either electronic monitoring using standard pill bottles fitted with microprocessors to record the time and frequency of bottle openings (Medication Event Monitoring System, MEMS); pill counts, comprising the number of pills left in a returned container; or questionnaires. Retrospectively collected data using pharmacy claims (de-identified data from administrative databases) were also noted. In studies with multiple data sources, the most sophisticated data source was recorded using the order MEMS > pill count > pharmacy claims data > questionnaire.

The relationship between compliance and patient outcome was investigated by recording clinical parameters (such as systolic or diastolic blood pressure, glycated haemoglobin levels and total blood cholesterol levels) and events (such as hospitalisations and emergency room visits). Cases were then classified according to the relationship between good compliance and persistence, and the change in outcome. For example, a positive relationship was taken as a positive change in outcome with good compliance, while a neutral relationship was taken as no change in outcome with good compliance.

### Statistical analysis

Results were presented in contingency tables. Associations between categorical variables were assessed using the chi-squared test and Fisher's exact test. Patient numbers and age, and study length, were compared using the *t*-test and Mann–Whitney test. The level of significance was taken as p < 0.05.

## Results

### Study characteristics

A total of 151 papers were identified from the literature search. A list of these papers is provided in the Appendix. From these papers, 139 studies which satisfied the inclusion criteria were identified, and were included in the analysis. Of the 139 studies, approximately one-third (31.6%) focused on hypertension, 27.3% on diabetes and 13.0% on dyslipidaemia. The remainder involved patients with CHD (17.3%) or CVD (10.8%). The majority of studies investigated one therapeutic class, and most were studies of AHTs (38.1%), followed by OADs (25.2%), LLTs (mostly statins; 23.0%) and multiple treatments (13.7%).

### Compliance definitions, measurement and data sources

The definitions and measures of compliance varied considerably between studies. Definitions were often not compatible with the standard MCP SIG definitions (Figure 1). Eighty-eight studies (63%) examined compliance only, 18 (13%) persistence only and 33 (24%) examined both compliance and persistence. The studies measuring both compliance and persistence were mostly retrospective in design, and were more likely to focus on LLTs (41%) than AHTs (17%), OADs (23%) or multiple treatments (10%).

Of the 139 studies, 104 (75%) used a continuous measure for compliance and 57 of these applied at least one cut-off value. The most common cut-off value applied to the MPR was 80% (43 studies).

**Figure 2:**
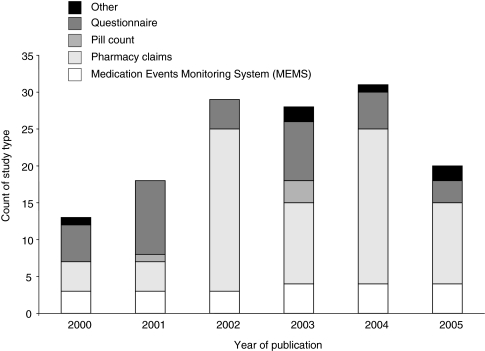
Source of compliance data over time

Analysis of the sources of compliance data showed that the largest number of studies used pharmacy claims data, followed by questionnaires, MEMS, ‘other’ sources and pill counts (Figure 2).

### Compliance

The most frequently reported measure of compliance was the 12-month MPR. The overall mean MPR was 72% [standard deviation (SD) 0.18], showing that only 72% of days ‘on therapy’ were actually covered by medication. The mean 12-month MPR did not differ significantly between therapeutic classes (Table 1). The overall proportion of patients with an MPR of > 80% was 59% (SD 0.19), showing that only 59% of patients had medication for more than 80% of their days ‘on therapy’ in the year. The proportion of patients with an MPR above 80% at 12 months was highest for AHTs (64%), followed by OADs (58%) and LLTs (51%), but the differences between therapeutic classes were not significant.

**Table 1:** Compliance results by therapeutic class, study design and data source

	Therapeutic class (%)				Study design (%)		Data source (%)	
			
Measure	AHTs (*n* = 53)	OADs (*n* = 35)	LLTs (*n* = 32)	Total* (*n* = 139)	Prospective (*n* = 65)	Retrospective (*n* = 54)	MEMS (*n* = 21)	Pharmacy claims (*n* = 73)
Average 12-month MPR	67 (12)	76 (10)	74 (8)	72 (34)	79 (5)	71 (29)	75 (4)	71 (29)
Proportion of patients withMPR > 80% at 12 months	64 (7)	58 (7)	51 (9)	59 (28)	67 (6)	57 (22)	65 (3)	57 (23)

Number of studies (*n*) are shown in parentheses.

* Includes studies with multiple treatment arms. AHTs, antihypertensives; LLTs, lipid-lowering therapies; MEMS, medication event monitoring system; MPR, medication possession ratio; OADs, oral antidiabetics.

### Persistence

**Figure 3:**
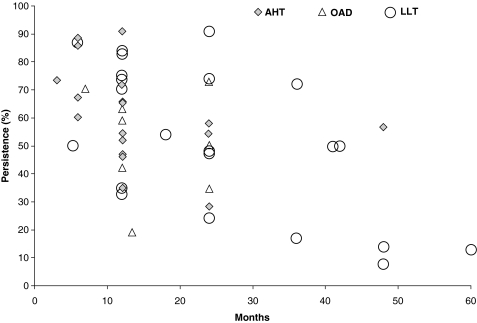
Persistence rates from the different studies, showing a significant trend (p < 0.001) towards decreased persistence with time

Many different measures of persistence were used over many different time frames. The 12-month persistence rate varied from 35.1% to 92.0% for the 22 estimates, with an average of 63.3% (SD 0.18). Persistence rates were similar for the different therapeutic classes (61.8% for AHTs, 62.3% for OADs and 65.6% for LLTs). There was a statistically significant trend towards decreased persistence with time (p < 0.001; Figure 3). The average persistence rate across the European studies was 61.7% over a mean observation period of 17 months. This compared with an average persistence rate of 51.1% in the US studies over a mean observation period of 21 months.

### Clinical outcomes

Fifty of the 139 studies (36%) reported outcomes. These comprised clinical parameters (e.g. systolic or diastolic blood pressure, blood glycated haemoglobin or cholesterol levels) or events (e.g. hospitalisations or emergency room visits). Therapeutic class did not appear to affect the relationship between compliance and outcome, whereas study design did; prospective studies were more likely than retrospective studies to show a relationship (p = 0.0001; Table 2).Studies using MEMS as a source of data were also significantly more likely to link compliance and outcome than studies using pharmacy claims (p = 0.004; Table 2).

**Table 2:** Percentage of studies reporting outcomes by therapeutic class, study design and data source

	Therapeutic class (%)				Study design** (%)		Data source*** (%)	
			
Outcome reported	AHTs (*n* = 53)	OADs (*n* = 35)	LLTs (*n* = 32)	Total* (*n* = 139)	Prospective (*n* = 65)	Retrospective (*n* = 54)	MEMS (*n* = 21)	Pharmacy claims (*n* = 73)
Clinical parameter	30.8	28.6	34.4	28.1	40.0	29.6	52.4	17.8
Event	7.7	5.7	9.4	7.9	12.3	5.6	14.3	4.1
None	61.5	65.7	56.3	64.0	47.7	64.8	33.3	78.1

Results are presented as percentages of the number of studies in each group.

* Includes studies with multiple treatment arms.

** p = 0.0001 for test of independence of factors.

*** p = 0.004 for test of independence of factors. AHTs, antihypertensives; LLTs, lipid-lowering therapies; MEMS, medication event monitoring system; OADs, oral antidiabetics.

The relationship between compliance and outcome was investigated in 41 of the 50 studies reporting outcomes. In 30 studies (73%), the effect of good compliance on outcome was positive, and a positive relationship was implied in a further three studies (7%). Only eight studies (20%) found good compliance to have a neutral or implied neutral effect on outcome.

### Study trends

An analysis of study trends according to the year of publication showed that the proportion of retrospective studies increased significantly between 2000 and 2005 (p = 0.002 for trend; Table 3). The use of continuous measures of compliance also increased significantly, while the use of discrete measures decreased (p = 0.009 for trend). Discrete measures are used most often in cross-sectional surveys and questionnaires, suggesting that use of such data sources became less common over the 5-year period. The average number of patients in each study, the mean patient age, the average study length, the distribution of studies across therapeutic class, the country of study and the source of funding did not change between 2000 and 2005.

**Table 3:** Study characteristics according to the year of publication

Publication		Study design (%)		Compliance measure (%)	
		
year	N	Prospective	Retrospective	Continuous	Discrete
2000	13	69	31	54	46
2001	18	78	22	50	50
2002	29	24	76	79	21
2003	28	57	43	82	18
2004	31	35	65	77	23
2005	20	40	60	90	10
Total	139	47	53	75	25
p-value for trend		0.002		0.009	

A comparison of retrospective and prospective studies showed that prospective studies were more likely to be European based, to involve fewer patients (mean 893 vs. 15,123 for retrospective studies; p = 0.001) and to be shorter in length (mean 12.5 months vs. 23.2 months for retrospective studies; p = 0.001) than retrospective studies. In addition, prospective studies were more likely to be funded by industry (54% funded by industry vs. 22% of retrospective studies; p = 0.00008).

### Therapeutic class

Patient numbers, the mean age of patients and the source of funding did not differ significantly between therapeutic classes. A higher proportion of LLT studies were retrospective (72%) compared with other therapeutic classes (40% for AHTs, 57% for OADs, 50% for multiple treatments), and there was a significant association between study design and therapeutic class (p = 0.042). LLT studies also showed the longest study duration (mean 30.1 months vs. 15.3 months for AHTs, 18.0 months for OADs, 10.7 months for multiple treatments). The mean study duration was significantly different between therapeutic classes (p = 0.001). Studies of multiple groups of treatments were shorter in duration than studies of a single group of medications (mean study duration 10.7 vs. 20.3 months respectively; p = 0.001).

## Discussion

The results of this study confirm the view that compliance and persistence with cardiovascular medication is poor, regardless of the method used for data collection (10, 11). In terms of compliance, patients filled 72% of prescriptions in the first year of treatment. Thus, almost 30% of days ‘on therapy’ were not actually covered by medication. Furthermore, only 59% of patients had medication for more than 80% of their days ‘on therapy’ in the year. The results of one study with a follow-up of more than 2 years showed that compliance decreases at first but then reaches a plateau (17).

Persistence also decreased with time, but with wide variability. The reasons for the variability in persistence rates are unclear. The 10% point difference between European and North-American persistence rates may have been due to differences in the average follow-up time between the different studies. However, other factors are clearly involved. In a study comparing compliance with statin therapy in Italy and Denmark, 91% of patients remained persistent after 2 years in Denmark, but only 48% remained persistent at this time in Italy (18). No single explanation for the difference emerged from the study, although differences in prescribing practices between the two countries could have played a role.

This systematic review confirmed previous findings that the definitions and measures of compliance vary considerably between studies (12, 13). However, there were some encouraging signs of a move towards using standard methodology, especially in retrospective analyses of pharmacy claims, in which MPR was the measure of choice in almost all cases. As studies move towards the use of a standard measure for compliance, it should become easier to compare estimates of compliance, enabling the influence of patient or regimen characteristics on compliance to be determined.

Many of the studies analysed in this review used the definition of MPR proposed by Steiner and Prochazka (16). It has been argued that it is more informative to report the percentage of patients above a certain threshold of MPR rather than mean MPR (19). However, the authors of this review believe that graphic presentation of the whole distribution as a histogram along with mean, SD or quintiles is preferable. Presenting compliance and persistence over time graphically is also very informative, as charted in 25 of the studies included in this review [see for example Sturkenboom et al. (20)].

The most important reason for investigating these issues is that poor compliance and lack of persistence with medications for hypertension, hyperlipidaemia and diabetes potentially lead to suboptimal health outcomes. Around one-third of studies in this review investigated the effect of compliance on outcomes, and the majority (73%) showed that compliance has a positive influence on outcome. Only one instance of a marginally negative effect of compliance on outcome (raised systolic blood pressure) was identified (21) and only a few studies found compliance to have a neutral effect on outcome (22–26). In addition, two of the studies reviewed (27, 28) found mortality rates to be lower in more compliant patients.

A number of recent studies have shown that patients who are compliant with therapy are likely to have better outcomes. In a meta-analysis, which included cardiovascular studies, a consistent association between good adherence to drug therapy and reduced mortality was found (29). Similarly, increased compliance and persistence with long-term cardiovascular therapy have been shown to reduce the risk of adverse cardiovascular outcomes; compliant patients (MPR ≥ 80%) showing a reduced predicted relative risk of 4.6% for men and 16.4% for women (11). Another study showed that patients who were highly compliant with antihypertensive therapy were 45% more likely to achieve blood pressure control than those with medium or low compliance (odds ratio 1.45, p = 0.026) (30).

In another study investigating the relationship between persistence (defined as < 60 days gap between refills) with antihypertensive therapy and the risk of MI/stroke, multivariate analysis showed that persistent users were at significantly lower risk of MI/stroke than non-persistent patients (relative risk 0.88, 95% confidence interval: 0.82–0.94) (31). Better compliance with antihypertensive therapy has also been shown to reduce the risk of hospitalisation (32), while better compliance with antidiabetic medication has been shown to reduce emergency room visits by 26% over a 2- to 3-year period (33).

Over the 5-year period of the present study, a trend towards more retrospective studies using data collected from pharmacy claims databases was seen. This was not surprising given that such studies take less time and money than prospective studies and potentially provide larger numbers of patients.

One limitation of the present review is that one person selected the studies and extracted the data. Thus, relevant studies may have been missed or incorrectly categorised. Another limitation is that although study characteristics and compliance measures were examined at the study level, differences between treatment arms within studies (e.g. different classes of AHTs, different doses) were not investigated. In studies with multiple treatment arms, population-weighted averages were used to calculate compliance. Meta-analysis was beyond the scope of this review. Because of the number of papers included in the review, study characteristics were presented only in tabular form. In addition, only means and SDs of compliance measures were calculated, without multivariate analysis. Finally, semi-transparency of definitions in some papers made it difficult to determine whether methods, and consequently compliance measures, were truly comparable.

Prospective real-world studies that use standard definitions and measures of compliance, and focus on objective outcomes, such as mortality, are needed to further our understanding of the issue of compliance. Large retrospective studies that analyse existing databases, identify appropriate stratification subgroups and use modelling exercises would also be useful.

## Conclusions

In this systematic review, poor compliance and persistence with cardiovascular and antidiabetic medication proved to be a significant problem, with almost 30% of days ‘on therapy’ not actually covered by medication and only 59% of patients having medication for more than 80% of their days ‘on therapy’ in the year. The definitions and measures of compliance/persistence used varied widely between studies making comparisons difficult. However, there were signs of a move towards the use of standard terminology and methodology. The most frequent measure of compliance was the 12-month MPR, which did not differ between therapeutic classes. Similarly, 12-month persistence rates did not differ between therapeutic classes but did show a significant trend towards a decrease over time. The majority of studies investigating the relationship between compliance and outcome found that compliance had a positive effect on outcome, suggesting that the management of CVD may be improved by improving patient compliance.

Further research into the problem of poor compliance with cardiovascular and antidiabetic medication is warranted to increase the number of published studies in this area and to increase awareness of the problem. By increasing awareness, it may be possible to improve patient compliance. The availability of different targeted interventions, including behavioural training and electronic devices designed specifically to improve patient compliance, may also contribute to improved compliance and persistence, and hence to improved clinical outcomes.

## References

[1] Dzau V, Braunwald E (1991). Resolved and unresolved issues in the prevention and treatment of coronary artery disease: a workshop consensus statement. Am J Heart.

[2] McGinnis JM, Foege WH (1993). Actual causes of death in the United States. JAMA.

[3] American Heart Association Heart Disease and Stroke Statistics – 2006 Update.

[4] Michaud CM, McKenna MT, Begg S (2006). The burden of disease and injury in the United States 1996. Popul Health Metr.

[5] Thom T, Haase N, Rosamond W (2006). Heart disease and stroke statistics – 2006 update. A Report from the American Heart Association Statistics Committee and Stroke Statistics Subcommittee.

[6] Prospective Studies Collaboration (1995). Cholesterol, diastolic blood pressure and stroke: 13,000 strokes in 450,000 people in 45 prospective cohorts. Lancet.

[7] Lewington S, Clarke R, Qizilbash N (2002). Age-specific relevance of usual BP to vascular mortality. Lancet.

[8] Baigent C, Keech A, Kearney PM, the Cholesterol Treatment Trialists’ (CTT) Collaborators (2005). Efficacy and safety of cholesterol-lowering treatment: prospective meta-analysis of data from 90,056 participants in 14 randomised trials of statins. Lancet.

[9] Cushman WC, Basile J (2006). Achieving blood pressure goals: why aren't we?. J Clin Hypertens (Greenwich).

[10] World Health Organization (WHO) (2003). Adherence to Long Term Therapies: Evidence for Action.

[11] Halpern MT, Khan ZM, Schmier JK (2006). Recommendations for evaluating compliance and persistence with hypertension therapy using retrospective data. Hypertension.

[12] Schedlbauer A, Schroeder K, Peters TJ (2004). Interventions to improve adherence to lipid lowering medication. Cochrane Database Syst Rev.

[13] Schroeder K, Fahey T, Ebrahim S (2004). Interventions for improving adherence to treatment in patients with high blood pressure in ambulatory settings. Cochrane Database Syst Rev.

[14] Berger ML, Bingefors K, Hedblom EC (2003). Healthcare Cost, Quality and Outcomes: ISPOR Book of Terms.

[15] ISPOR Medication Compliance and Persistence Special Interest Group (MCP): Accomplishments.

[16] Steiner JF, Prochazka AV (1997). The assessment of refill compliance using pharmacy records: methods, validity, and applications. J Clin Epidemiol.

[17] Chapman RH, Benner JS, Petrilla AA (2005). Predictors of adherence with antihypertensive and lipid-lowering therapy. Arch Intern Med.

[18] Larsen J, Vaccheri A, Andersen M (2000). Lack of adherence to lipid-lowering drug treatment. A comparison of utilization patterns in defined populations in Funen, Denmark and Bologna, Italy. Br J Clin Pharmacol.

[19] Wetzels GE, Nelemans P, Schouten JS (2004). Facts and fiction of poor compliance as a cause of inadequate blood pressure control: a systematic review. J Hypertens.

[20] Sturkenboom MCJM, Picelli G, Dieleman JP (2005). Patient adherence and persistence with antihypertensive therapy: one-versus two-pill combinations. J Hypertens.

[21] Choo PW, Rand CS, Inui TS, Lee ML, Ma CC, Platt R (2000). A pharmacodynamic assessment of the impact of antihypertensive non-adherence on blood pressure control. Pharmacoepidemiol Drug Saf.

[22] Bertholet N, Favrat B, Fallab-Stubi CL, Brunner HR, Burnier M (2000). Why objective monitoring of compliance is important in the management of hypertension. J Clin Hypertens (Greenwich).

[23] Blonde L, Wogen J, Kreilick C (2003). Greater reductions in A1C in type 2 diabetic patients new to therapy with glyburide/metformin tablets as compared to glyburide co-administered with metformin. Diabetes Obes Metab.

[24] Hope CJ, Wu J, Tu W, Young J, Murray MD (2004). Association of medication adherence, knowledge, and skills with emergency department visits by adults 50 years or older with congestive heart failure. Am J Health Syst Pharm.

[25] Rosen MI, Rigsby MO, Salahi JT (2004). Electronic monitoring and counseling to improve medication adherence. Behav Res Ther.

[26] Charpentier G, Fleury F, Dubroca I (2005). Electronic pill-boxes in the evaluation of oral hypoglycemic agent compliance. Diabetes Metab.

[27] Muhlestein JB, Horne BD, Bair TL (2001). Usefulness of in-hospital prescription of statin agents after angiographic diagnosis of coronary artery disease in improving continued compliance and reduced mortality. Am J Cardiol.

[28] Howell N, Trotter R, Mottram DR (2004). Compliance with statins in primary care. Pharm J.

[29] Simpson SH, Eurich DT, Majumdar SR (2006). A meta-analysis of the association between adherence to drug therapy and mortality. BMJ.

[30] Bramley TJ, Gerbino PP, Nightengale BS (2006). Relationship of blood pressure control to adherence with antihypertensive monotherapy in 13 managed care organizations. J Manag Care Pharm.

[31] Breekveldt-Postma NS, Siiskonen SJ, Penning-Van Beest FJA (2006). Non-persistent use of antihypertensive drugs leads to increased risk of hospitalizations for acute myocardial infarction or stroke. Value Health Suppl.

[32] Sokol MC, McGuigan KA, Verbrugge RR (2005). Impact of medication adherence on hospitalization risk and healthcare cost. Med Care.

[33] Mahoney JJ (2005). Reducing patient drug acquisition costs can lower diabetes health claims. Am J Manag Care.

